# The validity of proxy-based data on loneliness in suicide research: a case-control psychological autopsy study in rural China

**DOI:** 10.1186/s12888-018-1687-x

**Published:** 2018-05-01

**Authors:** Lu Niu, Cunxian Jia, Zhenyu Ma, Guojun Wang, Zhenjun Yu, Liang Zhou

**Affiliations:** 1 The Affiliated Brain Hospital of Guangzhou Medical University (Guangzhou Huiai Hospital), 36 Mingxin Road, Liwan District, Guangzhou, 510370 China; 2 School of Public Health, Shandong University, Jinan, China; 3 School of Public Health, Guangxi Medical University, Nanning, China; 4 Xiangya School of Public Health, Central South University, Changsha, China

**Keywords:** Loneliness, Psychometric properties, Psychological autopsy, Chinese rural elderly, Suicide

## Abstract

**Background:**

There is a lack of evidence for the role of loneliness on suicide using psychological autopsy method, and the validity of proxy informants’ reports on loneliness is not well established. This study aimed to investigate the validity of proxy respondent reports on loneliness, and the reliability and validity of the University of California Los Angeles Loneliness Scale-6 (ULS-6) as used in psychological autopsy method with rural elderly people in China.

**Methods:**

Two hundred forty-two suicide cases and 242 normal community controls were selected, and the psychological autopsy method was utilized to collect information. Data from proxy respondents of the living controls were compared with data reported by the targets (gold standards).

**Results:**

Subject-proxy concordance for ULS-6 was fair (ICC = 0.447) in the living controls. The suicide cases were more likely to have a higher score of ULS-6 than the living controls. Additionally, our data supported that ULS-6 had adequate psychometric properties in both suicide and control groups: factor analyses yielded one-factor component solution; Cronbach’s alpha (both > 0.90) demonstrated excellent internal consistency; the Spearman correlation analysis indicated that the ULS-6 score was positively correlated with depression; and negatively correlated with QOL and social support.

**Conclusions:**

Results support proxy-based data on loneliness in research of suicide in older adults in rural China, and the ULS-6 is a psychometrically sound instrument for measuring loneliness in psychological autopsy studies.

## Background

In China, a sharp decline in the official national suicide rate has been observed in the past two decades, from 15.6 per 100,000 in 2002 to 6.6 in 2015 [1]. However, the decline in suicide rate was less prominent in older age-groups compared with younger age-groups [1]. It was predicted that Chinese elderly suicide rates, especially among the rural elderly population, were likely to go up again [2]. Given the rapid aging of Chinese population, elderly suicide prevention should be given greater priority for reducing suicides in China [3]. To understand the causes and identify correlates of suicide, psychological autopsy, which gather data from postmortem proxy respondents, medical records, and other sources, is a widely used method [4, 5].

Loneliness is a feeling due to the lack of meaningful social relationships [6] or deficiency between actual and desired levels of social interaction [7]. Studies have linked loneliness to various correlates of suicidal behaviors. For example, loneliness was found as a significant predictor of depression and anxiety in the elderly [8, 9]. A high correlation was reported between loneliness and substance use as well [10]. Additionally, loneliness was found to be correlated with suicide ideation and suicide attempt [11–14]. A recent meta-analytic review found that older people experiencing loneliness were twice more likely to have suicidal ideation (OR = 2.24; 95%CI: 1.73-2.90) [15]. People suffering from loneliness also has a higher risk of suicide. In a content analysis of online suicide notes, loneliness was one of the core themes [16].

In psychological autopsy research, the supreme principle is that the greater the amount of relevant data analyzed, the more accurate the investigators’ conclusions are likely to be [17]. Though loneliness has been found to be associated with suicidal behaviors, there is a lack of evidence for the role of loneliness on suicide using psychological autopsy method. Besides, the validity of psychological autopsy studies depends on the validity of the information provided by proxy informants about the deceased [18]. Previous studies have suggested that proxies are good judges of past history of suicide attempts, suicidal intent, psychiatric diagnosis, depression, life events, and social support [4, 5, 19, 20]. As loneliness is a subjective and personal inner experience or feeling, is it amenable to detection by others? The validity of proxy informants’ reports on loneliness is not yet well established.

With different operationalizations of the construct of loneliness, there are several established instruments, such as the Rasch-Type Loneliness Scale [21], the Social and Emotional Loneliness Scale [22], and the University of California Los Angeles Loneliness Scale (ULS) [23]. The ULS with 20 items was firstly developed by Russell et al. in 1978 [23]. A four-item (ULS-4) and an eight-item (ULS-8) short form of ULS was later developed by Russell et al. [24] and Hays et al. [25], respectively. In 2002, Zhou et al. [26] has translated the ULS-8 into Chinese and revised the scale into 6 items (ULS-6). The Chinese version ULS-6 has shown good internal reliability, test-retest reliability, construct validity, and concurrent validity in Chinese rural community-dwelling elderly [26]. Thus, the ULS-6 was adopted to measure loneliness in this study.

In this case-control psychological autopsy study, we aimed to assess the concordance of subject and proxy reports on loneliness in the Chinese rural elderly; and to evaluate the psychometric properties of ULS-6 in individuals who died by suicide and, separately, in matched community-based control subjects in rural China.

## Methods

### Participants and procedures

This case-control psychological autopsy study was conducted, during June 2014 to September 2015, in Shandong Province, Hunan Province, and Guangxi Province. A total of 12 counties from the three provinces were randomly selected. In each county, suicide cases aged 60 and above were collected consecutively based on the death certification system. All village doctors and local public health professionals involved in death certification were given brief training and required to report all elderly suicide death to local Centers for Disease Control and Prevention (CDCs). After all relevant and available information was collected, the manner of death was determined by trained investigators.

For each suicide case, we randomly selected community living comparison based on age (±3 years old), gender, and living location. When a suicide case was identified, investigators would list and numerate all matched older adults. One living comparison was then selected from the list randomly by a computer program. Besides, for each suicide case and living comparison, we selected two informants: generally, the first informant was one next-to-kin who lived with the individual who died by suicide or the living comparison; and the second informant was always a friend, a neighbor, or a remote relative.

The information provided by two informants was combined as the proxy data for suicide cases and living comparisons. For demographic characteristics, information provided by the first informant was relied on. For each item of ULS-6, GDS, QOL, and DSSI, answers that were hypothetically associated with elevated risk of suicide were used, because a targeting symptom or behavior might exist as long as either informant had observed it. Thus, positive answer of an item of GDS was used when one of the two informants reported positive; similarly, higher score, and lower score of QOL and DSSI were used.

This study was approved by the Human Research Ethics Committees of Shandong University, Central South University, and Guangxi Medical University. Written informed consent has been obtained from participants in the living comparison group, and all informants of both suicide cases and living comparisons.

### Measurements

#### Demographic characteristics

Demographic data were collected such as gender, age, educational level, marital status, employment, and living arrangements. In this study, people that were married and living with spouse, or cohabiting were classified as “currently married”; while people with other marital status (including single, divorced, widowed, and married but living apart) were classified as “non-currently married”. Being left-behind was defined was during the last 12 months prior to death/investigation, all adult children had lived out of the original township for at least 10 months, and had visited their parents no more than twice.

#### University of California Los Angeles Loneliness Scale-6 (ULS-6)

Loneliness was assessed by the Chinese version of ULS-6. In 2002, the original ULS-8 was translated by Zhou et al. [26], and validated in elderly samples in a rural community. By exploratory factor analysis, a 6-item ULS (ULS-6) with one factor was generated. The ULS-6 has shown good internal reliability, test-retest reliability, construct validity, and concurrent validity in Chinese rural community-dwelling elderly [26].Each item is rated on a 4-point scale ranging from 1 (never) to 4 (often feel this way). The total score ranges from 6 to 24, with higher scores indicating higher levels of loneliness.

#### Geriatric Depression Scale (GDS)

Depression was measured by GDS, a 30-item screening instrument developed by Brink et al. [27]. It is a validated and reliable scale upon 30 yes-or-no questions that was developed to assess depressive symptoms in the past week among elderly people. Higher scores indicate higher levels of depression. A cut-off score of 10 and 20 represents mild and severe levels of depression, respectively. The GDS-30 has shown adequate properties in the Chinese elderly [28].

#### Quality of Life (QOL)

QOL was assessed by 6 items developed by Phillips et al. [29], regarding physical health, psychological health, economic circumstances, work, family relationships, and relationships with non-family associates in the past month. Each item is rated on a 5-point scale ranging from 1 (very poor) to 5 (excellent), with higher scores indicating better QOL.

#### Duke Social Support Index (DSSI)

Social support was measured by the 23-item DSSI [30]. It consists of three subscales: social interaction, subjective support, and instrumental support. The total score ranges from 11 to 45, with higher scores indicating higher levels of social support. The 23-item DSSI has shown good reliability and validity in Chinese elderly samples [31].

### Statistical analysis

First, in the living controls, proxy-based data were compared with subject-based data (gold standard). The Spearman correlation analysis, the pair-sample t-test, and intraclass correlation coefficients (ICC) were used. ICC values were evaluated based on the following benchmarks: poor, less than 0.40; fair, 0.40 − 0.59; good, 0.60 − 0.74; and excellent, greater than 0.75 [18, 32].

Then, the combined data were adopted to evaluate the psychometric properties of the ULS-6 for suicide cases and living controls, respectively. The exploratory factor analysis (EFA) was conducted to explore the underlying factor structure of the ULS-6. The sample adequacy was assessed by the Kaiser-Meyer-Olkin (KMO) test and Bartlet’s test. Internal reliability was assessed using the criterion of Cronbach’s alpha ≥0.70 and split-half reliability coefficient (Spearman-Brown coefficient) ≥ 0.70 [33, 34]. Independent-samples t-tests and one-way ANOVA were used for comparison among different demographic groups (gender, age groups, education, marital status, employment, living alone, and being left-behind). Concurrent validity was evaluated by correlations between the depression, quality of life, and social support.

All analyses were performed with SPSS version 23.0 (SPSS Inc., Chicago, IL, USA). Significance levels of this study were set at 0.05.

## Results

### Demographic characteristics

Altogether 242 suicide cases and 242 living comparisons were obtained. In the suicide cases, 43.8% were female, and the mean age was74.4 years (SD = 8.2). In the living controls, 43.8% were female, and the mean age was 74.1 years (SD = 8.2). As presented in Table 1, compared with the living controls, the suicide cases were more likely to be non-currently married, unemployed, living alone, and left-behind. Besides, suicides cases reported higher scores of depression, but lower scores of QOL and social support than the living controls.

**Table 1:** Demographic characteristics of suicide cases and living controls

Variable	Suicide cases (*n* = 242)	Living controls (n = 242)	*χ* ^*2*^ */t*	*P*
Gender			–	–
Male	135 (55.8)	135 (55.8)		
Female	107 (44.2)	107 (44.2)		
Age groups			0.913	0.633
60-69	73 (30.2)	70 (28.9)		
70-79	100 (41.3)	110 (45.5)		
≥ 80	69 (28.5)	62 (25.6)		
Education			1.920	0.383
Below primary school	111 (45.9)	96 (39.7)		
Primary school	105 (43.4)	116 (47.9)		
Above primary school	26 (10.7)	30 (12.4)		
Marital status			19.890	0.000
Currently married	122 (50.4)	170 (70.2)		
Non-currently married	120 (49.6)	72 (29.8)		
Employment			8.274	0.016
Employed	41 (16.9)	61 (25.2)		
Unemployed	194 (80.2)	167 (69.0)		
Retired	7 (2.9)	14 (5.8)		
Living alone			10.679	0.002
Yes	64 (26.4)	35 (14.5)		
No	178 (73.6)	207 (85.5)		
Being left-behind			4.491	0.046
Yes	41 (16.9)	25 (10.3)		
No	201 (83.1)	217 (89.7)		
Depression [mean (SD)]	21.40 (5.95)	9.22 (6.42)	21.654	0.000
QOL [mean (SD)]	15.40 (3.06)	19.50 (3.11)	14.629	0.000
Social support [mean (SD)]	22.88 (5.98)	27.47 (6.82)	7.868	0.000

### Subject-proxy agreement on loneliness

In the living controls, the mean score of ULS-6 reported by the subjects themselves was 9.8 (SD = 3.9). The mean score of ULS-6 from the proxy data was 11.0 (SD = 4.0). The Spearman correlation analysis indicated a low correlation between the self-reported and proxy data (*r* = 0.308, *p* = 0.000). The paired-sample T-test showed a significant difference (*t* = 4.045, *p* = 0.000). As shown in Table 2, there was moderate agreement on self-reported and proxy data of the ULS-6 based on the ICC value of 0.447 (*p* = 0.000). The consistency was low to moderate for each item, as the ICCs ranged from 0.295 to 0.503 (Table 3).

**Table 2:** Subject-proxy agreement on loneliness among living controls (n = 242)

Items of ULS-6	Self-reported data Mean (SD)	Proxy data Mean (SD)	Paired t-test	*p*	ICC	*p*
1. I lack companionship.	1.75 (0.94)	1.98 (0.92)	3.297	0.001	0.446	0.000
2. There is no one I can turn to.	1.66 (0.84)	1.85 (0.83)	2.913	0.004	0.372	0.000
3. I feel left out.	1.61 (0.77)	1.81 (0.82)	3.099	0.002	0.315	0.002
4. I feel isolated from others.	1.55 (0.72)	1.77 (0.75)	3.724	0.000	0.345	0.001
5. I am unhappy being so withdrawn.	1.52 (0.69)	1.72 (0.71)	3.418	0.001	0.295	0.003
6. People are around me but not with me.	1.67 (0.85)	1.85 (0.83)	2.849	0.005	0.503	0.000
The total score of ULS-6	9.76 (3.90)	10.99 (4.01)	4.045	0.000	0.447	0.000

**Table 3:** Factor matrix for the ULS-6 in suicide cases and living controls

Item description	Suicide cases (n = 242)		Living controls (n = 242)	
	Mean (SD)	Factor 1	Mean (SD)	Factor 1
1. I lack companionship.	2.75 (0.99)	0.849	1.98 (0.92)	0.793
2. There is no one I can turn to.	2.60 (0.94)	0.856	1.85 (0.83)	0.839
3. I feel left out.	2.67 (0.94)	0.916	1.81 (0.82)	0.880
4. I feel isolated from others.	2.52 (0.94)	0.898	1.77 (0.75)	0.875
5. I am unhappy being so withdrawn.	2.47 (0.90)	0.844	1.72 (0.71)	0.789
6. People are around me but not with me.	2.54 (0.99)	0.898	1.85 (0.83)	0.781
The total score of ULS-6	15.55 (5.00)		10.99 (4.01)	
Eigenvalue		4.619		4.106
Proportion of explained variance (%)		76.991		68.425

### Construct validity

In the suicide cases, the KMO value was 0.910, and the Bartlett’s test of Sphericity was statistically significant (χ^2^ = 1245.214, *p* = 0.000), indicating the adequacy of proceeding with factor analysis. The EFA results yielded an one-factor solution that accounted for 66.092% of the variance. Item loadings ranged from 0.844 to 0.916 (Table 3).

In the living controls, the KMO value was 0.871, and the Bartlett’s test of Sphericity was statistically significant (χ^2^ = 921.187, *p* = 0.000), indicating the adequacy of proceeding with factor analysis. The EFA results yielded an one-factor solution that accounted for 68.425% of the variance. Item loadings ranged from 0.781 to 0.880.

### Reliability

In the suicide cases, the Cronbach’s α for ULS-6 was 0.940. The Spearman-Brown coefficient was 0.919.

In the living controls, the Cronbach’s α for ULS-6 was 0.905. The Spearman-Brown coefficient was 0.878.

### Convergent validity

In the suicide cases, the mean score of ULS-6 was 15.55 (SD = 5.00), which was significantly higher than that of the living controls (*t* = 11.054, *p* = 0.000).

As shown in Table 4, people, who had non-currently married, were living alone, and were left-behind, were more likely to report higher ULS-6 scores in both suicides and living controls. There was no significant difference on the ULS-6 scores between different groups of gender, age groups, education, and employment (*p* > 0.05). As presented in Table 5, the Spearman correlation analysis indicated that the ULS-6 score was significantly and positively correlated with depression; and negatively correlated with QOL and social support (all *p* < 0.01).

**Table 4:** Demographic factors related to loneliness in suicides cases and living controls

	Suicide cases (n = 242)			Living controls (n = 242)		
Variable	ULS-6 Mean (SD)	*t*	*P*	ULS-6 Mean (SD)	*t*	*P*
Marital status		3.539	0.000		3.925	0.000
Currently married	14.44 (5.20)			10.35 (3.78)		
Non-currently married	16.67 (4.55)			12.50 (4.15)		
Living alone		5.082	0.000		3.565	0.000
Yes	18.14 (4.47)			13.17 (4.50)		
No	14.61 (4.86)			10.62 (3.81)		
Being left-behind		2.587	0.010		3.125	0.002
Yes	17.37 (5.21)			13.32 (4.34)		
No	15.17 (4.89)			10.72 (3.89)		

There was no significant difference on the ULS-6 score between different groups of gender, age groups, education, and employment (*p* > 0.05)

**Table 5:** Correlations of the ULS-6 to validating scales in suicide cases and living controls

Scale	Suicide cases (n = 242)	Living controls (n = 242)
	ULS-6 (Spearman’s rho)	ULS-6 (Spearman’s rho)
GDS score	0.277^a^	0.516^a^
QOL score	−0.344^a^	-0.382^a^
DSSI score	-0.443^a^	-0.466^a^

^a^Correlation is significant at the 0.01 level

## Discussion

The aim of this study was to assess whether information derived without input from the targets themselves was valid for identifying the feeling of loneliness using a Chinese version of ULS-6 in rural China. In this case-control psychological autopsy study, we used the data of the living control group to compare the information from proxy respondents with the data reported by the targets themselves (gold standard). The agreement was fair between the living targets and proxy respondents about the degree of loneliness with an ICC value of 0.447. This finding was consistent with previous research on factors related to internal and emotional experience; the subject-proxy agreement on internal emotional experience were found to be moderate or poor, such as anxiety [4] and perceived emotional support [5, 19].

The estimates of the proxy respondents were significantly and slightly higher than subject reports with about a mean difference of 1.23 (SD = 4.72). Symptoms and behaviors that are prominent, noticeable, and recurrent are the most detectable [20]. With increasing age, social networks of older adults will become smaller (e.g., loss of spouse and friends), and social contracts will decrease as well due to worsen physical health and reduced physical activity performance. Thus, from external perception, proxy respondents may easily overestimate the subject’s feeling of loneliness. Still, the significant correlation coefficients between the proxy respondents and the living targets themselves indicate comparatively accurate estimates of the proxy respondents on the reality of the loneliness feeling of the targets.

The information bias could exist in both suicide cases and living controls. The case-control design could minimize the effect of information bias. By comparison of proxy data between suicide cases and living controls, it could better estimate the role of risk factors for suicide [35]. In the suicide cases, the mean score of ULS-6 was significantly higher than that of the living controls, which indicates that loneliness should be considered as a risk factor of suicide in Chinese rural elderly.

Moreover, this study presents the psychometric characteristics of the ULS-6 as used in psychological autopsy method. Exploratory Factor Analysis was adopted to assess the construct validity of the ULS-6, and the one-factor structure was found for both suicide and control groups. The reliability findings supported that the ULS-6 has good internal consistency with the Cronbach’s α value over 0.90 in both suicide and control groups. It was similar to the previous study conducted in the elderly in rural China [26].

The association between social support and loneliness has been showed in abundant studies that a high level of social support can reduce the feeling of loneliness [36–38]. The results of the present study are consistent with the previous findings. People, who were non-currently married, living alone, and left behind by children, and had a lower score of social support, were more likely to report higher ULS-6 scores in both suicide and control groups. Emotional loneliness could be caused by the absence of a secure attachment person in one’s life such as a spouse [37], and close relationships are long-term predictors of loneliness in older age [38]. Rural elderly people that live alone, are non-currently married, and left behind by children, should be evaluated as a high-risk group. Interventions targeted social support systems, especially intimate relationships are needed to alleviate loneliness feeling among the rural elderly in China.

The concurrent validity was additionally evaluated by correlations between the ULS-6 and depression and QOL. As mentioned above, many elderly people experienced loneliness either as a result of living alone, a lack of close family ties or social support. It is not surprising to find a positive correlation between loneliness and depression in both suicide and control groups. A similar correlation was reported in the previous literature [8, 9, 39]. Our study also indicated that there was a significant correlation between loneliness and QOL in both suicide and control groups. In other words, the QOL decreases along with increased feelings of loneliness, which is also supported by previous evidence [37, 40]. Thus, the construct validity of the ULS-6 was supported and demonstrates the expected link between loneliness and depression and QOL in both suicide and control groups.

There are some limitations. In this study, we used the data from the community normal living controls as the gold standard to assess the validity of their informants’ responses. As suggested in previous studies [5, 19], the informants of suicide cases are most likely to be in grief, which may influence their responses to the information of the deceased. It might be asymmetrical to generalize the response from the non-grieving normal people in the living controls. Future studies may need to find ways to adjust this imbalance. Further, this study focuses on rural elderly. Future study of this type should be conducted with other age groups, and continue to evaluate its psychometric properties in psychological autopsy studies.

## Conclusions

This study helps to establish the validity of proxy respondent based data on loneliness in research on suicide in older adults in rural China. Proxy respondents are generally fair judges of targets’ loneliness. The ULS-6, with adequate reliability and validity, can be used in psychological autopsy research to collect data that may be ultimately inform prevention efforts, and the role of loneliness in suicide should be further studied. In addition, the ULS-6 shows potential for identifying elderly people that may be at risk for suicide. Rural elderly people that live alone, are left behind by children, non-currently married, and have higher depression and poor quality of life, are more likely to suffer from loneliness, and should be evaluated as a high-risk group.

## Abbreviations

CDC: Center for Disease Control and Prevention
DSSI: Duke Social Support Index
EFA: Exploratory factor analysis
GDS: Geriatric Depression Scale
ICC: Intraclass correlation coefficient
KMO: Kaiser-Meryer-Olkin test
QOL: Quality of life
SD: Standard deviation
ULS-6: University of California Los Angeless Loneliness Scale with 6 items

## References

[1] Liu Z, Huang Y, Ma C, Shang L, Zhang T, Chen H. Suicide rate trends in China from 2002 to 2015. Chin Ment Health J. 2017;31(10):756–67.

[2] Zhong BL, Chiu HF, Conwell Y (2016). Elderly suicide trends in the context of transforming China, 1987–2014. Sci Rep.

[3] Zhang D, Yang Y, Sun Y, Wu M, Xie H, Wang K, Zhang J, Jia J, Su Y (2017). Characteristics of the Chinese rural elderly living in nursing homes who have suicidal ideation: a multiple regression model. Geriatr Nurs.

[4] Conner KR, Duberstein PR, Conwell Y (2001). The validity of proxy-based data in suicide research: a study of patients 50 years of age and older who attempted suicide. I. Psychiatric diagnoses. Acta Psychiatr Scand.

[5] Zhang J, Conwell Y, Wieczorek WF, Jiang C, Jia S, Zhou L (2003). Studying Chinese suicide with proxy-based data: reliability and validity of the methodology and instruments in China. J Nerv Ment Dis.

[6] Fees BS, Martin P, Poon LW (1999). A model of loneliness in older adults. J Gerontol.

[7] Peplau LA, Perlman D, Peplau LA, Perlman D. Blueprint for a social psychological theory of loneliness. Love Attraction. 1979:101–10.

[8] Ahmed D, El Shair IH, Taher E, Zyada F (2014). Prevalence and predictors of depression and anxiety among the elderly population living in geriatric homes in Cairo, Egypt. J Egypt Public Health Assoc.

[9] Singh A, Misra N (2009). Loneliness, depression and sociability in old age. Ind Psychiatry J.

[10] Segrin C, McNelis M, Pavlich CA. Indirect effects of loneliness on substance use through stress. Health Commun. 2018:33(5)513–8. 10.1080/10410236.2016.1278507 28157378

[11] Minayo MC, Cavalcante FG (2015). Suicide attempts among the elderly: a review of the literature (2002/2013). Cien Saude Colet.

[12] Goodman ML, Serag H, Keiser PK, Gitari S, Raimer BG (2017). Relative social standing and suicide ideation among Kenyan males: the interpersonal theory of suicide in context. Soc Psychiatry Psychiatr Epidemiol.

[13] Chang EC, Wan L, Li P, Guo Y, He J, Gu Y, Wang Y, Li X, Zhang Z, Sun Y (2017). Loneliness and suicidal risk in young adults: does believing in a changeable future help minimize suicidal risk among the lonely?. J Psychol.

[14] Sharma B, Lee TH, Nam EW. Loneliness, insomnia and suicidal behavior among school-going adolescents in western Pacific Island countries: role of violence and injury. Int J Environ Res Public Health 2017,14(7):791. 10.3390/ijerph14070791 PMC5551229 28714893

[15] Chang Q, Chan CH, Yip PSF (2017). A meta-analytic review on social relationships and suicidal ideation among older adults. Soc Sci Med.

[16] Synnott J, Ioannou M, Coyne A, Hemingway S: A Content Analysis of Online Suicide Notes: Attempted Suicide Versus Attempt Resulting in Suicide. Suicide Life Threat Behav 2017. 10.1111/sltb.12398. [Epub ahead of print] 10.1111/sltb.12398 28960422

[17] Knoll JL (2008). The psychological autopsy, part I: applications and methods. J Psychiatr Pract.

[18] An J, Phillips MR, Conner KR (2010). Validity of proxy-based reports of impulsivity and aggression in Chinese research on suicidal behavior. Crisis.

[19] Conner KR, Conwell Y, Duberstein PR (2001). The validity of proxy-based data in suicide research: a study of patients 50 years of age and older who attempted suicide. II. Life events, social support and suicidal behavior. Acta Psychiatr Scand.

[20] Conner KR, Beautrais AL, Brent DA, Conwell Y, Phillips MR, Schneider B (2011). The next generation of psychological autopsy studies. Part I. Interview content. Suicide Life Threat Behav.

[21] Jonggierveld JD, Kamphuls F (1985). The development of a Rasch-type loneliness scale. Appl Psychol Measur.

[22] Ditommaso E, Spinner B (1993). The development and initial validation of the social and emotional loneliness scale for adults (SELSA). Personal Individ Differ.

[23] Russell D, Peplau LA, Ferguson ML (1978). Developing a measure of loneliness. J Pers Assess.

[24] Russell D, Peplau LA, Cutrona CE (1980). The revised UCLA loneliness scale: concurrent and discriminant validity evidence. J Pers Soc Psychol.

[25] Hays RD, Dimatteo MR (1987). A short-form measure of loneliness. J Pers Assess.

[26] Zhou L, Li Z, Hu M, Xiao S (2012). Reliability and validity of ULS-8 loneliness scale in elderly samples in a rural community. J Cent South Univ (Medical Sciences).

[27] Brink TL, Yesavage JA, Lum O, Heersema PH, Adey M, Rose TL (1982). Screening tests for geriatric depression. Clin Gerontol.

[28] He X, Xiao S, Zhang D (2008). Reliability and validity of the Chinese version of geriatric depression scale: a study in a population of Chinese rural community-dwelling elderly. Chin J Clin Psych.

[29] Phillips MR, Yang G, Zhang Y, Wang L, Ji H, Zhou M (2002). Risk factors for suicide in China: a national case-control psychological autopsy study. Lancet.

[30] Koenig HG, Westlund RE, George LK, Hughes DC, Blazer DG, Hybels C (1993). Abbreviating the Duke social support index for use in chronically ill elderly individuals. Psychosomatics.

[31] Mao H, Qiu P, Wen P, Yang Y, Hao T, Gao X, Yuan P (2015). Reliability and validity of DSSI-23 scale in a rural elderly population. J Sichuan Univ (Medical Science Edition).

[32] Cicchetti DV (1994). Guidelines, criteria, and rules of thumb for evaluating normed and standardized assessment instruments in psychology. Psychol Assess.

[33] Niu L, Qiu Y, Luo D, Chen X, Wang M, Pakenham KI, Zhang X, Huang Z, Xiao S (2016). Cross-culture validation of the HIV/AIDS stress scale: the development of a revised Chinese version. PLoS One.

[34] Hu Y, Zhang Z, Xie J, Wang G (2017). The outpatient experience questionnaire of comprehensive public hospital in China: development, validity and reliability. International J Qual Health Care.

[35] Isometsa ET (2001). Psychological autopsy studies--a review. Eur Psychiatry.

[36] Wang G, Zhang X, Wang K, Li Y, Shen Q, Ge X, Hang W (2011). Loneliness among the rural older people in Anhui, China: prevalence and associated factors. Int J Geriatr Psychiatry.

[37] Arslantaş H, Adana F, Abacigil EF, Kayar D, Acar G (2015). Loneliness in elderly people, associated factors and its correlation with quality of life: a field study from western Turkey. Iran J Public Health.

[38] Dahlberg L, Andersson L, Lennartsson C (2018). Long-term predictors of loneliness in old age: results of a 20-year national study. Aging Ment Health.

[39] van den Brink RH, Schutter N, Hanssen DJ, Elzinga BM, Rabeling-Keus IM, Stek ML, Comijs HC, Penninx BW, Oude Voshaar RC. Prognostic significance of social network, social support and loneliness for course of major depressive disorder in adulthood and old age. Epidemiol Psychiatr Sci. 2017:1–12. 10.1017/S2045796017000014 PMC6998855 28183368

[40] Chalise HN, Saito T, Takahashi M, Kai I (2007). Relationship specialization amongst sources and receivers of social support and its correlations with loneliness and subjective well-being: a cross sectional study of Nepalese older adults. Arch Gerontol Geriatr.

